# Effectiveness of cauda epididymal plasma-2 and lecithin based diluents to minimize abnormality of sexing albumin spermatozoa during cold storage

**DOI:** 10.14202/vetworld.2021.2543-2548

**Published:** 2021-09-27

**Authors:** Frediansyah Firdaus, Dian Ratnawati

**Affiliations:** Department of Animal Physiology and Reproduction, Beef Cattle Research Institute, Pasuruan East Java 67184, Indonesia.

**Keywords:** Lecithin, cauda epididymal plasma-2, glutathione, spermatozoa abnormality

## Abstract

**Background and Aim:**

Lecithin based diluent such as AndroMed, is a semen diluent made without animal components to prevent the risk of disease transmission, while glutathione (GSH) is an intracellular non-enzymatic antioxidant that prevents cell damage due to reactive oxygen species. Meanwhile, the specific impact of AndroMed and GSH combinations on spermatozoa abnormalities has not been fully studied. Therefore, this study aims to determine the effect of using cauda epididymal plasma-2 (CEP-2) and AndroMed diluents with or without the addition of GSH on the abnormalities of sexing semen of Ongole crossbred bulls in cold storage.

**Materials and Methods:**

This study used a factorial completely randomized design 2×2, the first factor was types of diluent and the second was with or without the addition of GSH. Observation of spermatozoa abnormalities was carried out at a storage time of 0-5 days using 297 ejaculations of liquid semen, with 100 spermatozoa observed per smear of each ejaculate. Furthermore, the data were analyzed using a two-way analysis of variance and the significant threshold (p-value) for statistical analysis was set at <0.05.

**Results:**

The results showed that there was a significant difference (p<0.05) between AndroMed and CEP-2 in minimizing the abnormalities of upper layer spermatozoa (X), with parameters DH and AD on day 0, damage of spermatozoa (DMR) on days 1-5, and dag-like defect (DLD) on day 5. Furthermore, spermatozoa abnormalities in the lower layer (Y) showed a significant difference between diluents in the parameters of AD on day 1, DMR on days 0-5, and DLD on days 1-5. The significant difference between with or without the addition of GSH in the X sperm was observed in the DH parameters on day 0 and DMR on 5, while there was no significant difference in the Y sperm.

**Conclusion:**

Based on the results, AndroMed has the potential to minimize spermatozoa abnormalities compared to CEP-2 diluent in sexed liquid semen. Therefore, AndroMed diluents with or without the addition of 1 mM GSH have no significant effect on spermatozoa abnormalities.

## Introduction

Breeding soundness evaluation (BSE) was introduced to identify the fertility of bulls as breeders with the requirement of having spermatozoa abnormalities <30% [1]. Boe-Hansen *et al*. [2] stated that spermatozoa abnormalities correlated with DNA integrity and protamine deficiency, which have the potential of harming embryo development due to DNA and ­chromatin damage. Moreover, artificial insemination technology with sexing spermatozoa is useful in determining the sex of calves based on maintenance goals. Meanwhile, sexing methods that have been developed include Percoll gradient, Sephadex and BSA columns, flow cytometry, and albumin gradient [3]. Several studies have been conducted on sexing albumin gradient because the materials and ­technicalities are ­relatively easy. The principle of separating with an albumin gradient is based on the motility speed, where spermatozoa with high motility of carrying Y chromosomes penetrate earlier to the more concentrated albumin separating media [4].

Cauda epididymal plasma (CEP) is a semen diluent developed by Verberckmoes *et al*. [5] by mimicking the conditions of epididymal cauda of bull. A previous study by Ducha *et al*. [6] showed that CEP-2 with 20% egg yolk maintains the ultrastructure of the spermatozoa membrane for 8 days at a temperature of 4-5°C to protect its fertility. Moreover, lecithin based diluent such as AndroMed, is a semen diluent made without animal components such as egg yolk to prevent the influence of unwanted hormones, bacteria, and drug residues [7]. It protects the spermatozoa membrane during the sexing process to minimize abnormalities [8]. Meanwhile, glutathione (GSH) is an intracellular non-enzymatic antioxidant that prevents cell damage due to reactive oxygen species (ROS). The addition of 0.5-1 mM of GSH maintains the quality of liquid semen buffalo to the 5^th^ day. Furthermore, GSH improves motility, viability, membrane integrity, reduces membrane damage, and increases the fertility of buffalo semen [9].

Therefore, this study aims to determine the effect of using CEP-2 and AndroMed semen diluents, with or without GSH on the abnormalities of sexed crossbreed Ongole (OC) bulls at different storage times.

## Materials and Methods

### Ethical approval

All experiments were approved by the Indonesian Agency for Agricultural Research and Development (IAARD) animal ethics committee (Balitbangtan/Lolitsapi/Rm/01/2018).

### Study period and location

This study was conducted from July 2018 to June 2019 using liquid semen samples of 297 ejaculates from four Ongole crossbred (OC) bulls (3-6 years old) with spermatozoa motility ≥70% [10]. The semen was obtained at the Experimental Housing, Beef Cattle Research Institute, Grati, Pasuruan, at a temperature of 20-22°C. Furthermore, processing of liquid semen, sexing, and spermatozoa abnormalities observation was carried out in the Laboratory of Reproduction, Beef Cattle Research Institute.

### Semen processing

Spermatozoa sexing was carried out using the 5%, 10%, and 15% albumin gradient method [3]. The spermatozoa were stored in the refrigerator at a temperature of 3-5°C from day 0 to 5 (0, 1, 3, and 5), with a concentration of 100×10^6^ m/L using CEP-2 and AndroMed with or without the addition of 1 mM GSH. Meanwhile, the CEP-2 was based on the development of Verberckmoes *et al*. [5] methods.

### Observation of spermatozoa abnormality

The observation of spermatozoa abnormalities was carried out on days 0, 1, 3, and 5 using Sperm Class Analyzer Microptics v. 2.1 (Barcelona, Spain). Moreover, examination of spermatozoa abnormalities started with the making of a semen smear with a glass object. Furthermore, one drop of semen was mixed with a drop of eosin-nigrosin stain, stirred with ose, reviewed, and was fixed on the fire. The semen smear was examined using a 1000×microscope. Furthermore, the identification and calculation of each type of abnormality were carried out on approximately 100 spermatozoa, and the percentage was based on the following formula:


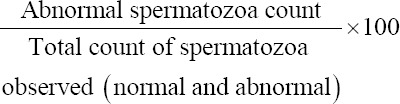


Spermatozoa abnormality observed were large and small abnormal head [10], dag-like defect (broken midpiece) [11], pyriform head [12], nuclear vacuoles, distal midpiece reflex [13], detached head [14], acrosome defect, simple bent tail, and terminally coiled tail [15].

### Statistical analysis

This study used a factorial completely randomized design 2×2, the first factor was the types of diluent (CEP-2 and AndroMed) and the second was an addition of (with or without) GSH. The data were analyzed using the two-way analysis of variance with IBM SPSS Statistics 23 and the significant threshold (p-value) for statistical analysis was set at <0.05.

## Results

### Spermatozoa in upper layer (X)

The results showed that the different types of diluents (CEP-2 and AndroMed) with or without the addition of GSH showed no significant differences (p>0.05) in the parameters of subarachnoid hemorrhage (SAH), luteinizing hormone (LH), pyriform head (PH), nuclear vacuoles (NV), spontaneous breathing trial (SBT), and tracheostomy collar trial (TCT) (Table-1) spermatozoa in the top layer (X) during cold storage. Furthermore, significant differences in GSH treatment were shown on DH storage on day 0 and damage of spermatozoa (DMR) on day 5. The duration of storage of spermatozoa from sexing albumin on day 0 showed that CEP-2 minimizes the damage to DH spermatozoa (p<0.05) compared to AndroMed. Moreover, AndroMed showed a significant difference (p<0.05) in minimizing the damage to AD abnormalities compared to CEP-2 on day 0, while storage for days 1, 3, and 5 showed no significant difference (p>0.05).

**Table-1:** Abnormalities of spermatozoa in the upper layer (X) resulting from sexing albumin during cold storage.

Parameter	Day	Diluent (D)		GSH (G)		Significance		
		
		CEP-2	AndroMed	With	Without	D	G	D*G
Head								
Detached head	0	1.33±0.88	2.67±1.87	3.00±1.73	1.15±0.80	*	*	*
	1	1.42±1.83	2.46±1.76	1.67±1.55	2.23±2.08	ns	ns	ns
	3	0.79±0.97	1.21±1.12	1.14±1.02	0.86±1.09	ns	ns	ns
	5	0.55±0.68	1.75±1.28	1.38±1.40	0.82±0.87	ns	ns	ns
Small abnormal head	0	0.25±0.62	0.00±0.00	0.18±0.60	0.08±0.27	ns	ns	ns
	1	0.17±0.38	0.31±0.48	0.25±0.45	0.23±0.43	ns	ns	ns
	3	0.07±0.26	0.00±0.00	0.00±0.00	0.07±0.26	ns	ns	ns
	5	0.00±0.00	0.00±0.00	0.00±0.00	0.00±0.00	ns	ns	ns
Large head	0	0.17±0.38	0.00±0.00	0.09±0.30	0.08±0.27	ns	ns	ns
	1	0.17±0.38	0.08±0.27	0.08±0.28	0.15±0.37	ns	ns	ns
	3	0.00±0.00	0.14±0.53	0.00±0.00	0.14±0.53	ns	ns	ns
	5	0.00±0.00	0.13±0.35	0.00±0.00	0.09±0.30	ns	ns	ns
Pyriform head	0	0.00±0.00	0.00±0.00	0.00±0.00	0.00±0.00	ns	ns	ns
	1	0.08±0.28	0.00±0.00	0.08±0.28	0.00±0.00	ns	ns	ns
	3	0.00±0.00	0.00±0.00	0.00±0.00	0.00±0.00	ns	ns	ns
	5	0.09±0.30	0.13±0.35	0.00±0.00	0.18±0.40	ns	ns	ns
Nuclear vacuoles	0	0.00±0.00	0.00±0.00	0.00±0.00	0.00±0.00	ns	ns	ns
	1	0.00±0.00	0.15±0.55	0.00±0.00	0.15±0.55	ns	ns	ns
	3	0.07±0.26	0.00±0.00	0.07±0.26	0.00±0.00	ns	ns	ns
	5	0.00±0.00	0.00±0.00	0.00±0.00	0.00±0.00	ns	ns	ns
Acrosome defect	0	0.67±1.15	0.00±0.00	0.64±1.20	0.08±0.27	*	ns	ns
	1	0.25±0.45	0.23±0.59	0.25±0.45	0.23±0.59	ns	ns	ns
	3	0.14±0.36	0.21±0.42	0.14±0.36	0.21±0.42	ns	ns	ns
	5	0.09±0.30	0.00±0.00	0.00±0.00	0.09±0.30	ns	ns	ns
Midpiece								
Distal midpiece reflex	0	15.83±2.65	11.92±7.36	13.18±4.57	14.46±6.76	ns	ns	ns
	1	18.25±3.13	10.08±6.03	15.50±6.81	12.62±5.76	*	ns	ns
	3	16.93±6.40	9.07±4.90	15.00±7.39	11.00±5.92	*	ns	ns
	5	24.55±5.06	7.25±5.39	20.88±9.89	14.64±9.90	*	*	ns
Dag-like defect	0	1.50±2.46	2.33±2.87	2.45±2.62	1.46±2.69	ns	ns	ns
	1	1.58±1.73	1.31±1.25	1.33±1.30	1.54±1.67	ns	ns	ns
	3	1.36±2.24	0.71±1.54	1.57±1.86	0.50±1.87	ns	ns	ns
	5	2.64±2.20	0.38±0.51	1.50±1.92	1.82±2.18	*	ns	ns
Tail								
Simple bent tail	0	0.08±0.28	0.00±0.00	0.09±0.30	0.00±0.00	ns	ns	ns
	1	0.08±0.28	0.00±0.00	0.00±0.00	0.08±0.27	ns	ns	ns
	3	0.00±0.00	0.00±0.00	0.00±0.00	0.00±0.00	ns	ns	ns
	5	0.00±0.00	0.00±0.00	0.00±0.00	0.00±0.00	ns	ns	ns
Terminally coiled tail	0	0.67±0.88	1.25±1.28	0.82±1.16	1.08±1.11	ns	ns	ns
	1	0.42±0.69	0.15±0.37	0.42±0.69	0.15±0.37	ns	ns	*
	3	0.29±0.61	0.14±0.53	0.36±0.74	0.07±0.26	ns	ns	ns
	5	1.64±2.29	0.50±1.06	1.50±2.23	0.91±1.64	ns	ns	ns

Values are mean±standard deviation. GSH=Glutathione, CEP: Cauda epididymal plasma, ns=non-significant,

* =significant (p<0.05)

AndroMed has the potential to minimize the morphological DMR compared to CEP-2 (Table-1) at the storage of days 1, 3, and 5 (p<0.05), while storage on day 0 has no significant difference (p>0.05). The morphological damage of DMR spermatozoa using CEP-2 on day 5 was approximately 24.55%, while AndroMed was able to maintain the morphological damage of storage on day 5 with abnormalities below 15%. In addition, AndroMed minimizes the damage of DLD morphology spermatozoa compared to CEP-2 (p<0.05) on day 5, while the storage on days 0, 1, and 3 showed no significant differences between the types of diluents (p>0.05).

### Spermatozoa in lower layer (Y)

The results showed that different diluents (CEP-2 and AndroMed) show no significant differences (p>0.05) in the parameters of DH, SAH, LH, PH, NV, SBT, and TCT lower layer spermatozoa (Y) (Table-2). Similarly, the addition (with or without) GSH gave no significant difference in all storage time and abnormal spermatozoa parameters. The difference in the use of different diluents affected the AD value in the lower layer of spermatozoa (Y) on day, while the results of storage on days 0, 3, and 5 showed no significant difference.

**Table-2:** Abnormalities of spermatozoa in the lower layer (Y) resulting from sexing albumin during cold storage.

Parameter	Day	Diluent (D)		GSH (G)		Significance	
		
		CEP-2	AndroMed	With	Without	D	G	D*G
Head								
Detached head	0	2.13±1.72	1.71±0.99	2.23±1.58	1.69±1.25	ns	ns	ns
	1	1.08±1.44	1.13±1.06	1.23±1.42	1.00±1.03	ns	ns	ns
	3	1.14±1.46	1.62±1.55	1.46±1.66	1.29±1.38	ns	ns	ns
	5	0.91±1.22	1.67±1.43	1.58±1.37	1.00±1.34	ns	ns	ns
Small abnormal head	0	0.07±0.25	0.07±0.26	0.00±0.00	0.13±0.34	ns	ns	ns
	1	0.25±0.45	0.27±0.79	0.46±0.87	0.07±0.26	ns	ns	ns
	3	0.07±0.26	0.00±0.00	0.00±0.00	0.07±0.26	ns	ns	ns
	5	0.09±0.30	0.08±0.28	0.17±0.38	0.00±0.00	ns	ns	ns
Large head	0	0.00±0.00	0.00±0.00	0.00±0.00	0.00±0.00	ns	ns	ns
	1	0.00±0.00	0.33±0.61	0.23±0.59	0.14±0.36	ns	ns	ns
	3	0.00±0.00	0.15±0.55	0.15±0.55	0.00±0.00	ns	ns	ns
	5	0.09±0.30	0.33±0.65	0.17±0.38	0.27±0.64	ns	ns	ns
Pyriform head	0	0.07±0.25	0.07±0.26	0.08±0.27	0.06±0.25	ns	ns	ns
	1	0.00±0.00	0.00±0.00	0.00±0.00	0.00±0.00	ns	ns	ns
	3	0.00±0.00	0.08±0.27	0.00±0.00	0.07±0.26	ns	ns	ns
	5	0.00±0.00	0.00±0.00	0.00±0.00	0.00±0.00	ns	ns	ns
Nuclear vacuoles	0	0.00±0.00	0.00±0.00	0.00±0.00	0.00±0.00	ns	ns	ns
	1	0.00±0.00	0.00±0.00	0.00±0.00	0.00±0.00	ns	ns	ns
	3	0.07±0.26	0.00±0.00	0.08±0.27	0.00±0.00	ns	ns	ns
	5	0.00±0.00	0.00±0.00	0.00±0.00	0.00±0.00	ns	ns	ns
Acrosome defect	0	0.07±0.25	0.00±0.00	0.08±0.27	0.00±0.00	ns	ns	ns
	1	0.42±0.51	0.07±0.25	0.23±0.43	0.21±0.42	*	ns	ns
	3	0.00±0.00	0.08±0.27	0.08±0.27	0.00±0.00	ns	ns	ns
	5	0.00±0.00	0.00±0.00	0.00±0.00	0.00±0.00	ns	ns	ns
Midpiece								
Distal midpiece reflex	0	14.93±3.28	11.07±4.96	14.77±4.18	11.69±4.48	*	ns	ns
	1	19.58±4.94	8.40±5.93	13.62±9.36	13.14±6.45	*	ns	ns
	3	19.93±5.42	6.23±4.43	14.69±7.83	12.07±9.20	*	ns	ns
	5	23.00±8.43	8.42±3.65	17.08±10.05	13.55±9.47	*	ns	ns
Dag-like defect	0	3.27±6.91	1.43±2.27	3.08±7.53	1.81±2.13	ns	ns	ns
	1	1.17±1.85	1.20±1.82	1.77±2.24	0.64±1.08	ns	ns	ns
	3	0.79±1.18	0.15±0.37	0.54±1.12	0.43±0.75	ns	ns	ns
	5	1.55±1.44	0.50±0.67	1.25±1.48	0.73±0.78	*	ns	*
Tail								
Simple bent tail	0	0.00±0.00	0.07±0.26	0.08±0.27	0.00±0.00	ns	ns	ns
	1	0.00±0.00	0.07±0.25	0.00±0.00	0.07±0.26	ns	ns	ns
	3	0.07±0.26	0.00±0.00	0.08±0.27	0.00±0.00	ns	ns	ns
	5	0.18±0.40	0.08±0.28	0.17±0.38	0.09±0.30	ns	ns	ns
Terminally coiled tail	0	0.47±0.91	0.93±1.14	0.69±1.10	0.69±1.01	ns	ns	ns
	1	0.25±0.86	0.80±2.30	0.46±0.87	0.64±2.40	ns	ns	ns
	3	0.29±0.61	0.38±0.50	0.38±0.65	0.29±0.46	ns	ns	ns
	5	1.18±1.32	0.25±0.62	0.92±1.31	0.45±0.82	ns	ns	ns

Values are mean±standard deviation. GSH=Glutathione, CEP: Cauda epididymal plasma, ns=non-significant,

* =significant (p<0.05)

AndroMed showed significantly different results (p<0.05) compared to CEP-2 in minimizing DMR damage in the lower layer (Y) spermatozoa from sexing albumin during cold storage on days 0, 1, 3, and 5 (Table-2). Moreover, CEP-2 maintains DMR damage to a maximum of 23%, while AndroMed keeps DMR damage to a maximum of 12%. Furthermore, AndroMed and CEP-2 showed significant differences (p<0.05) at storage on day 5 with reduced damage <2% for both types of diluent.

## Discussion

The process of sexing and cold storage (3-5°C) increases the percentage of abnormal spermatozoa. Moreover, damage to the spermatozoa morphology can be an indication of cell damage that influences its fertility. Fitzpatrick *et al*. [16] stated that spermatozoa abnormalities were consistently associated with calf output; therefore, morphological assessment needs to be included in the BSE program to ensure that bulls have at least 70% normal sperm and not only on mass or individual motility.

**Figure-1:**
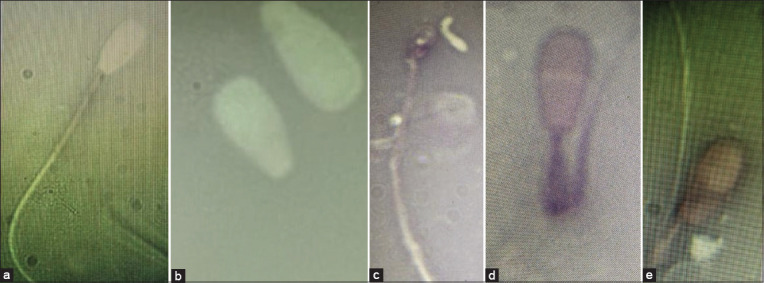
(a) normal, (b) detached head, (c) acrosome defect, (d) distal midpiece reflex, and (e) dag-like defect.

The most common abnormality of spermatozoa in the upper (X) and lower (Y) layer from sexing albumin during cold storage was the secondary morphological damage in the form of DMR. Similarly, Ghirardosi *et al*. [17] stated that DMR was the most common type of abnormality in bull, followed by PH, DH, and DLD. Distal midpiece reflex (Figure-1) is the damage of spermatozoa with features resembling a circular tail with cytoplasmic droplets in a circle caused by contact with a hypotonic solution [13]. It is also caused by an increase in superoxide dismutase due to the shock reaction of spermatozoa stored at cold temperatures [18].

A DLD (Figure-1) is the damage of the midpiece in the form of backward bending of the tail [19]. Moreover, the incidence of DLD occurred from the centrifugation process in the sexing process of spermatozoa. Furthermore, the presence of DMR and DLD abnormalities showed that 1 mM GSH administration cannot protect spermatozoa from damage during cold storage. Abnormalities in the midpiece are very detrimental because it is the place for the mitochondria organ that converts the ATP and ADP into energy for spermatozoa tail motility to hinder its.

The detached head (Figure-1) is a condition where the spermatozoa heads are removed from the tail and the incidence rate is usually less than 10%, in association with testicular hypoplasia. Meanwhile, the high incidence of DH is an indication of hereditary traits [20]. This abnormality is caused by an error during the sexing process or sample preparation. Furthermore, sexing using egg white albumin through several stages such as physical treatment by centrifugation contributes to spermatozoa abnormalities.

Acrosome defects (Figure-1) are described as refractile or dark stained areas on the sperm head [21] that reduces the ability of spermatozoa to penetrate the zona pellucida [14]. The morphological defects in AD are often characterized by indented sperm apex [22]. Meanwhile, spermatozoa with AD morphological damage have impaired plasma membrane function which causes premature capacity and spontaneity of acrosome reactions during incubation after thawing. Furthermore, abnormalities in the spermatozoa head have a significant effect on fertility because its plays an important role in penetrating the zona pellucida and binding with the ovum [14].

During cold storage (3-5°C), spermatozoa continue to carry out metabolic activities which lead to byproducts ROS that damages the spermatozoa. Moreover, longer shelf life produces higher ROS which leads to an increase in the percentage of abnormal spermatozoa. Kusumawati *et al*. [23] stated that an increase in spermatozoa abnormalities occur due to the separation process of sexing, centrifugation, cold storage, and washing which requires high energy to maintain spermatozoa physiological condition.

The significant difference in spermatozoa abnormalities that occurred in the liquid semen from sexing the upper (X) and lower layer (Y) spermatozoa showed that there is a direct relationship between differences in diluents and the spermatozoa abnormality. Meanwhile, AndroMed minimizes spermatozoa abnormalities compared to CEP-2 due to the ingredient of phospholipid lecithin which is based on soybeans [24]. A previous study showed that AndroMed diluent produces better spermatozoa quality compared to animal protein-based extenders [25]. The extender composition has an important role as a medium to protect spermatozoa in the preservation process. Murphy *et al*. [26] stated that commercial plant-based extenders such as AndroMed provide substantial advantages. Meanwhile, egg yolk-based diluents make the microscopic assessment of semen more difficult, especially when using CASA assistance [27], and potentially have a risk of exotic diseases transmission such as flu birds [28].

## Conclusion

AndroMed minimizes spermatozoa abnormalities compared to CEP-2 diluent. Therefore, AndroMed diluents with or without the addition of 1 mM GSH have no significant effect on spermatozoa abnormalities.

## Authors’ Contributions

FF: Collected data, wrote the original manuscript, and reviewed the final version of the manuscript. DR: Designed the study, collected and analyzed data, and reviewed the final version of the manuscript. Both authors read and approved the final manuscript.
